# Urethra-sparing stereotactic body radiotherapy for prostate cancer: how much can the rectal wall dose be reduced with or without an endorectal balloon?

**DOI:** 10.1186/s13014-018-1059-1

**Published:** 2018-06-19

**Authors:** Angèle Dubouloz, Michel Rouzaud, Lev Tsvang, Wilko Verbakel, Mikko Björkqvist, Nadine Linthout, Joana Lencart, Juan María Pérez-Moreno, Zeynep Ozen, Lluís Escude, Thomas Zilli, Raymond Miralbell

**Affiliations:** 1 Geneva University Hospital, Rue Gabrielle-Perret-Gentil 4, 1205 Genève, Switzerland; 2 Department of Radiation Oncology, Chaim Sheba Medical Center, Tel-Hashomer, 52621 Ramat Gan, Israel; 3 Department of Radiation Oncology, VU medical center, De Boelelaan 1117, P.O. Box 7057, 1007 MB Amsterdam, The Netherlands; 4 Department of Oncology and Radiotherapy, Turku University Hospital, PO Box 52, 20521 Turku, Finland; 5 Department of Medical Physics, Turku University Hospital, PO Box 52, 20521 Turku, Finland; 6 Onze-Lieve-Vrouw Ziekenhuis, Moorselbaan 164, 9300, Aalst, Belgium; 7 Serviço de Radioterapia Externa, Instituto Portugues de Oncologia, Rua Dr Antonio Bernardino de Almeida, 4200-072 Porto, Portugal; 8 Servicio de Oncología Radioterápica, Centro Integral Oncológico “Clara Campal”, Hospital Universitario Madrid Sanchinarro, C/ Oña 10, 28050 Madrid, Spain; 9 Neolife Medical Center, Nisbetiye Mah. Yucel Sokak, No: 6 Besiktas, 34340 Istanbul, Turkey; 10 Servei de Radiooncologia, Institut Oncològic Teknon, C/ Vilana 12, 08022 Barcelona, Spain; 11 Radiation Oncology Department, Geneva University Hospital, CH-1211, 14 Geneva, Switzerland

**Keywords:** Stereotactic body radiotherapy, Endorectal balloon, Dosimetric optimization, Prostate cancer, Urethra sparing

## Abstract

**Background:**

This is a dosimetric comparative study intended to establish appropriate low-to-intermediate dose-constraints for the rectal wall (R_wall_) in the context of a randomized phase-II trial on urethra-sparing stereotactic body radiotherapy (SBRT) for prostate cancer. The effect of plan optimization on low-to-intermediate R_wall_ dose and the potential benefit of an endorectal balloon (ERB) are investigated.

**Methods:**

Ten prostate cancer patients, simulated with and without an ERB, were planned to receive 36.25Gy (7.25Gyx5) to the planning treatment volume (PTV) and 32.5Gy to the urethral planning risk volume (uPRV). Reference plans with and without the ERB, optimized with respect to PTV and uPRV coverage objectives and the organs at risk dose constraints, were further optimized using a standardized stepwise approach to push down dose constraints to the R_wall_ in the low to intermediate range in five sequential steps to obtain paired plans with and without ERB (Vm_1_ to Vm_5_). Homogeneity index for the PTV and the uPRV, and the Dice similarity coefficient (DSC) for the PTV were analyzed. Dosimetric parameters for R_wall_ including the median dose and the dose received by 10 to 60% of the R_wall_, bladder wall (B_wall_) and femoral heads (F_Heads_) were compared. The monitor units (MU) per plan were recorded.

**Results:**

Vm_4_ reduced by half D_30%_, D_40%_, D_50%,_ and D_med_ for R_wall_ and decreased by a third D_60%_ while HI_PTV_, HI_uPRV_ and DSC remained stable with and without ERB compared to Vm_ref_. HI_PTV_ worsened at Vm_5_ both with and without ERB. No statistical differences were observed between paired plans on R_wall_, B_wall_ except a higher D_2%_ for F_heads_ with and without an ERB.

**Conclusions:**

Further optimization to the R_wall_ in the context of urethra sparing prostate SBRT is feasible without compromising the dose homogeneity to the target. Independent of the use or not of an ERB, low-to-intermediate doses to the R_wall_ can be significantly reduced using a four-step sequential optimization approach.

## Background

Stereotactic body radiotherapy (SBRT) for prostate cancer is emerging as a safe treatment option for patients with localized disease [1]. Clinical interest in extreme hypofractionated treatments results from estimated low α/β ratio values for prostate tumors (i.e. ≈1.5Gy) compared with the nearby organs at risk (OAR); rectum, urethra and bladder, with α/β values of 3–5 Gy [2, 3]. A treatment strategy, such as intentionally under-dosing areas of potentially lower tumor burden, for example the periurethral transitional zone of the prostate, may be used to reduce the risk of radiation induced urinary toxicity [4–6]. However, the risk of rectal toxicity is a major concern when designing prostate dose-escalation studies. Both conventionally fractionated radiotherapy and SBRT studies have shown that higher dose to the rectum correlates with increased rectal toxicity [7]. Minimizing the dose to the rectum, over the whole range from low to high doses, could impact the quality of life [8].

Guidelines for dose constraints for OAR for prostate SBRT are either inexistent or not well established. For the most widely used SBRT schedule (7.25 Gy, 5 fractions), recommended dose constraints have been published by King et al. [1]. These are the constraints that have been adopted by the *Novalis Circle Phase II Trial* (*ClinicalTrials.gov* *Identifier NCT01764646*). Dose to the rectum can be reduced by using either a software-based technique i.e. dosimetry plan optimization with the goal of reducing the dose to the rectal wall (R_wall_) [9], and/or gadget-based techniques, such as the use of an inflated endorectal balloon (ERB) [10] or prostate-rectal spacers [11–13].

We aimed to perform a dosimetric comparative study undertaken in the context of a randomized phase-II trial on urethra-sparing SBRT for prostate cancer. The principal goal of the study was to determine the optimal strategy for minimizing the low-to-intermediate-dose regions of the R_wall_, with the secondary goal of assessing the potential dosimetric benefit for the R_wall_ of using an ERB compared to no-ERB.

## Methods

Ten prostate cancer patients (cT1-3a N0 M0, Roach index for lymph-node involvement < 20%), treated between February 2013 and June 2014, were selected for the study. They were part of a population of 170 patients recruited in nine countries as part of a prospective multicentric randomized phase-II trial of short vs. protracted urethral-sparing SBRT for localized prostate cancer. This study concerns the first 10 consecutive patients recruited in one of the institution. They were all simulated, planned, and treated with an ERB inflated with 100 ml air [14].

For each patient, a first computed tomography simulation scan (CT_sim_) was acquired with an ERB, followed by a second CT_sim_ without the ERB. Planning CTs were acquired with axial slices of 2-mm thickness. A pediatric urinary catheter was introduced for accurate urethra delineation. A rigid registration with a pelvic magnetic resonance imaging (MRI) acquired on a flat table with the patient in the same treatment position and with an ERB was performed for definition of clinical target volume (CTV) and urethra in the CT_sim_ dataset acquired with ERB. On the other hand, the diagnostic MRI, realized without ERB, was used for contouring purposes for the second CT_sim_ dataset acquired without ERB.

A rectal enema was performed at home or in the clinic, based on patient preference, at the CT_sim_ and during the treatment course prior to insertion of the ERB (6 times in total). The enema was well tolerated overall and if necessary a second enema was performed if the rectum was not completely emptied. Patients were positioned in the supine position and immobilized with the *Combifix™* system. They were instructed to drink 600–700 mL of water one hour before the procedure, immediately after emptying their bladders. Three to four fiducials were implanted transrectally in the prostate under ultrasound guidance by an experienced uro-radiologist a minimum of seven days prior to the CT_sim_ acquisition for image-guidance.

The planning risk volume for the urethra (uPRV) consisted of the urethra plus a surrounding isotropic expansion of 3 mm inside the transitional zone (maximum axial uPRV size 1 cm). The CTV included the prostate, with or without the seminal vesicles (6 and 4 patients, respectively). The planning target volume (PTV) was defined as the CTV plus a 5 mm isotropic expansion in all directions except posteriorly, where a 3 mm expansion was used, excluding the uPRV. The absolute volumes of the PTV with and without ERB were similar for each patient: median 96.4 cm^3^ (range, 68.0–143.6) with the ERB and 92.4 cm^3^ (range 57.0–131.7) without. A 3 mm thick R_wall_ was defined on each CT from the lowest level of the ischial tuberosities to the rectosigmoid flexure. A 5 mm thick bladder wall (B_wall_) and both proximal femurs (F_heads_) were contoured on the corresponding CT axial slices. All contours were drawn in the Eclipse (Varian Medical Systems, Palo Alto, USA) treatment planning system (TPS) version 10 by the same radiation oncologist following the male pelvis normal tissue RTOG consensus contouring guidelines [15].

Plans were optimized with the progressive resolution optimizer (PRO v10.0.28 in Eclipse) and calculated with the analytical anisotropic algorithm (AAA v10.0.28). The treatment was delivered in two full volumetric modulated arcs (VMAT) with 6 MV beams using an accelerator equipped with a 2.5 mm leaf width HDMLC. The SBRT protocol prescribed 36.25 Gy in 5 fractions to the PTV, with a dose limit of 32.5 Gy to the uPRV, resulting in a biologically equivalent dose in 2 Gy per fraction (EQD2) of approximately 90 Gy to the PTV (α/β = 1.5Gy) and 62 Gy to the uPRV (α/β = 3). The plan normalization goal aimed to achieve 98% of the PTV receiving 95% of the prescribed dose (D_98%_ = 34.4Gy) with a maximum of 2% of PTV receiving no more than 107% of the prescribed dose (D_2%_ ≤ 38.8Gy). Similarly, the goal for the uPRV was D_98%_ ≥ 30.9 Gy (95% of 32.5Gy) and D_2%_ ≤ 35.8 Gy (107% of 32.5Gy). Dose constraints for the R_wall_ were V_36.25 Gy_ < 5%, V_32.6 Gy_ < 10% V_29 Gy_ < 20% (Table 1); for the B_wall_ the constraints were V_36.25 Gy_ < 10%, V_32.6 Gy_ < 20%, and V_18.1 Gy_ < 50%; while for the F_heads_ the constraint was D_2%_ ≤ 18.1 Gy.

**Table 1:** Original dose-constraints for R_wall_ as used for the paired reference plans (Vm_ref_) and the proposed additional dose-constraints based on the paired plans Vm_4_

Volume	Original dose-constraints (Vmref)	Additional dose-constraints after optimization (Vm4)
Rwall	V36.25 Gy < 5%	V13.1 Gy ≤ 30%
	V32.6 Gy < 10%	V7.2 Gy ≤ 40%
	V29 Gy < 20%	Dmed ≤6.5 Gy

For each patient a pair of reference plans (Vm_ref_) was created: one with ERB and one without ERB. The reference plans were optimized in order to respect the PTV and uPRV coverage objectives and the OAR dose constraints, without any additional sparing on the R_wall_ in the low or intermediate dose range. The optimization parameters were adapted separately on an individual basis for each patient/ERB combination to produce the reference plans. Then the intermediate-and-low dose sparing of the R_wall_ was forced further by adding three additional dose-volume optimization (DVO) constraints on the R_wall_ (D_2%_, D_10%_ and D_15%_) and by decreasing their value at each steps. These constraints were assigned a constant weight. Their initial values were set to start in the intermediate dose range (D_2%_ = 18Gy, D_10%_ = 15Gy and D_15%_ = 13Gy) down to low dose range (6, 3 and 1Gy, respectively) for the last optimization step. The additional DVO constraints were similar with and without ERB and applied for each patient. This resulted in a total of 120 plans (12 plans per patient - 6 with ERB and 6 without ERB) ranging from the reference plans (Vm_ref_), optimized with the initial dose constraints, to extreme optimization plans (Vm_1_ to Vm_5_), optimized with additional DVO objectives on the R_wall_. The homogeneity index for the PTV (HI_PTV_) and the uPRV (HI_uPRV_) was determined using: (D_2%_–D_98%_)**/**D_50%_. The Dice Similarity Coefficient (DSC) between the PTV and the volume encompassed by the 95% isodose line (V_95%_) was calculated as the ratio of their intersection and their union. DSC = (V_95%_∩PTV)**/(**V_95%_∪ PTV). Dosimetric parameters for the PTV, uPRV, and OAR were calculated. In addition, the median dose (D_med_) to the R_wall_, as well as the dose received by 10 to 60% of the R_wall_ volume, in increments of 10% (D_10%_ to D_60%_), were analyzed. The total number of monitor units (MU) was recorded.

The comparison of the dosimetric parameters with and without ERB at each step of the optimization was performed using the *Friedman* non-parametric analysis of variance. *Post-hoc* tests were performed according to the *Dunn-Bonferroni* procedure, with *p-values* adjusted for multiple comparisons. Statistics were computed using SPSS version 22; *p-values* ≤ 0.05 were considered statistically significant (two-sided tests).

## Results

**Fig. 1:**
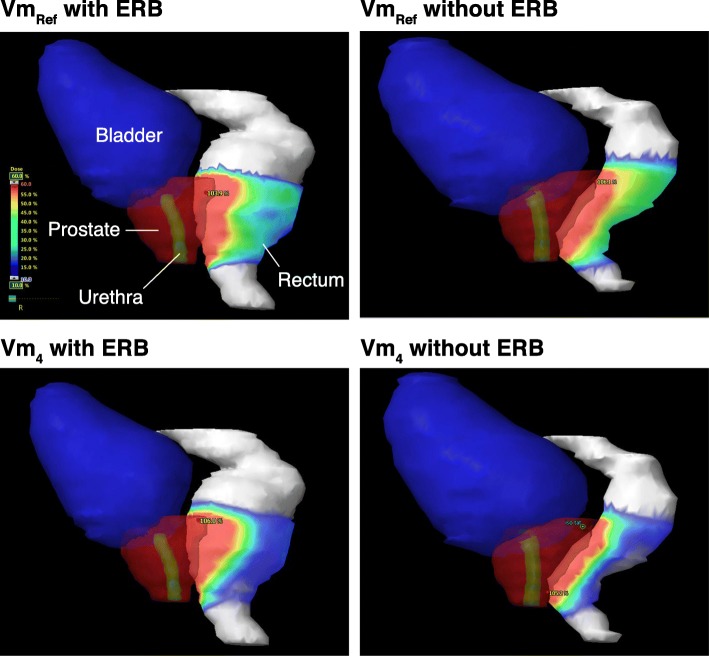
Three-dimensional representation of the PTV, uPRV, bladder, and rectum for the same patient with a colorwash display of the dose (from 10 to 60%) comparing plans Vm_ref_ vs. Vm_4_ with and without ERB

Figure 1 displays the dose distribution in a lateral projection of a reconstructed PTV, bladder and rectum, with and without ERB, in non-optimized and optimized treatment plans (Vm_ref_ and Vm_4_), respectively. It illustrates that the low-to-intermediate dose to the R_wall_ (3.3–21.8 Gy, i.e. 10–60% of the prescription dose 36.25 Gy) was strongly reduced using the plan optimization strategy in a similar way with and without ERB.

**Fig. 2:**
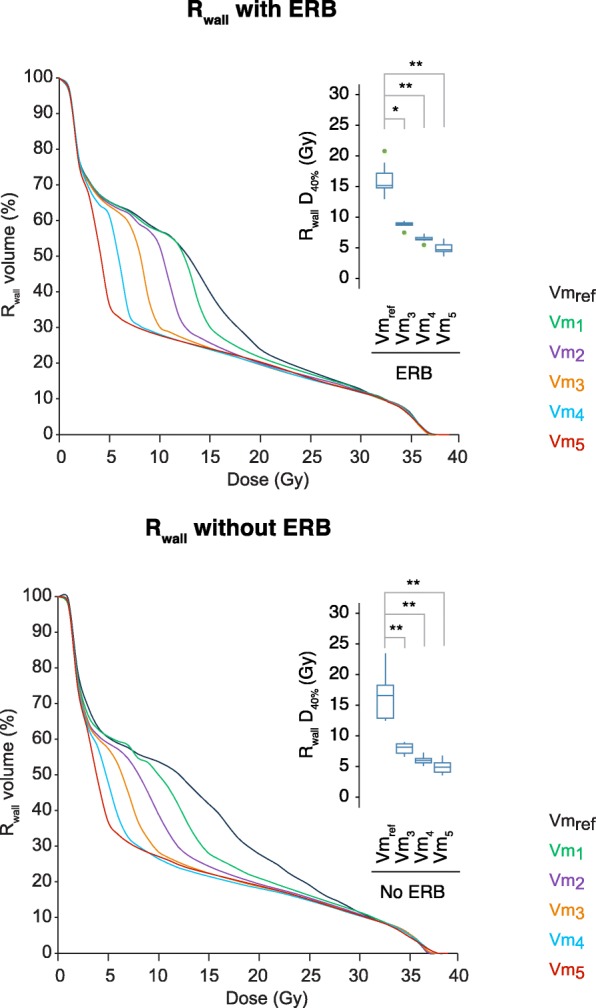
Dosimetric parameters for R_wall_. Median DVH from Vm_ref_ to Vm_5_ with ERB and without ERB. Box-and-whisker plots for Vm_ref_, Vm_3_, Vm_4_, and Vm_5_ of D_40%_. Significant relations are shown with gray lines above the boxplots. * is set for P <  0.05 and ** for P <  0.005. Outliers are visible with green dots for out-values (1.5xIQR)

Figure 2 shows the R_wall_ dose-volume histogram (DVH), with a focus on D_40%,_ for plans from Vm_ref_ to Vm_5_ with and without ERB. The optimization strategy achieves a significant decrease of low-to-intermediate dose to the R_wall_. Step Vm_4_ halved D_30%_, D_40%_, D_50%,_ and D_med_ and decreased by a third D_60%_ compared to Vm_ref_ (Table 2).

**Table 2:** Dosimetric data for PTV, uPRV and OAR with and without ERB. Comparison between the treatment plans Vm_ref_ and Vm_4_

	ERB			No ERB		
	Vmref	Vm4	*P-value*	Vmref	Vm4	*P-value*
PTV						
HI (u.a.)	0.096 [0.071–0.119]	0.099 [0.082–0.121]	0.721	0.086 [0.066–0.102]	0.096 [0.076–0.105]	0.156
DSC (u.a.)	0.779 [0.714–0.898]	0.773 [0.689–0.906]	1	0.810 [0.738–0.948]	0.795 [0.698–0.89]	1
D2% (Gy)	38.0 [37–38.9]	38.1 [37.4–39]	0.433	37.6 [36.8–38.2]	38.0 [37.2–38.3]	0.276
uPRV						
HI (u.a.)	0.088 [0.051–0.13]	0.092 [0.06–0.121]	1	0.099 [0.057–0.139]	0.098 [0.063–0.199]	1
Rwall						
D10% (Gy)	32.6 [29.9–35.8]	32.4 [25.6–35.7]	0.229	31.8 [29.9–35.6]	30.9 [26.7–35.3]	0.172
D20% (Gy)	23.1 [18.2–28.8]	19.8 [10.7–27.6]	< 0.001	24.7 [19.2–29.5]	19.2 [14.4–28.5]	< 0.001
D30% (Gy)	17.9 [15–22.9]	8.5 [6.2–13.1]	< 0.001	19.6 [14.5–24.9]	8.3 [6.1–13.7]	< 0.001
D40% (Gy)	15.2 [13–20.8]	6.5 [5.5–7.3]	< 0.001	16.6 [12.5–23.5]	6.0 [5.1–7.3]	< 0.001
D50% (Gy)	12.9 [4.2–19.6]	6.0 [3.3–6.8]	< 0.001	12.8 [6.3–22.2]	5.0 [4.1–6.5]	< 0.001
D60% (Gy)	9.1 [2.5–18.2]	5.4 [2.2–6.4]	< 0.001	6.1 [2.4–19.7]	4.1 [2.2–5.8]	0.041
Dmed (Gy)	12.9 [4.1–19.5]	5.9 [3.3–6.7]	< 0.001	12.2 [6.3–22.1]	4.9 [4–6.5]	< 0.001
Bwall						
V18.1 Gy (%)	32.6 [15.6–47.5]	28.5 [13.3–45.5]	0.010	26.0 [14.9–44.8]	23.2 [13.1–41.8]	0.036
Fheads						
D2%	10.6 [9–14.1]	16.3 [13.7–17.8]	< 0.001	11 [8.3–12.6]	13.3 [10.7–16.5]	0.141
MU	2110 [1788–2478]	2897 [2515–3168]	< 0.001	2256 [1834–2897]	2763 [2419–3161]	0.002

**Fig. 3:**
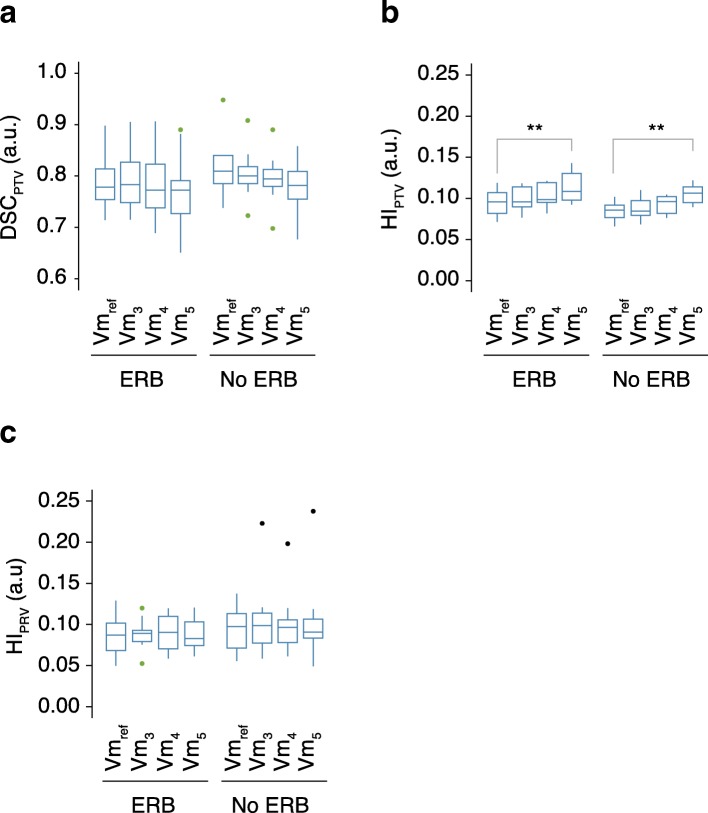
Box-and-whisker plots of (A) DSC_PTV_, (B) HI_PTV_ and (C) HI_uPRV_ with ERB and without ERB for Vm_ref_, Vm_3_, Vm_4_, and Vm_5_. Outliers are visible with green dots for out-values (1.5xIQR) and black dots for extreme out-values (3xIQR). Significant relations are shown with gray lines above the boxplots. * is set for P <  0.05 and ** for P <  0.005

The extreme optimization objectives on the R_wall_ worsen the target homogeneity index HI_PTV_ (*p-value <  0.01*) in a similar way with and without ERB in Vm_5_, because of a higher D_2%_ (*p* <  0.01): 38.5 Gy vs 38.0Gy with ERB; 38.4Gy vs 37.6Gy without ERB. Both the DSC and the HI_uPRV_ remained stable (Fig. 3). No differences were observed in terms of DSC, HI_PTV_, and HI_uPRV_, when comparing paired plans with and without ERB.

**Fig. 4:**
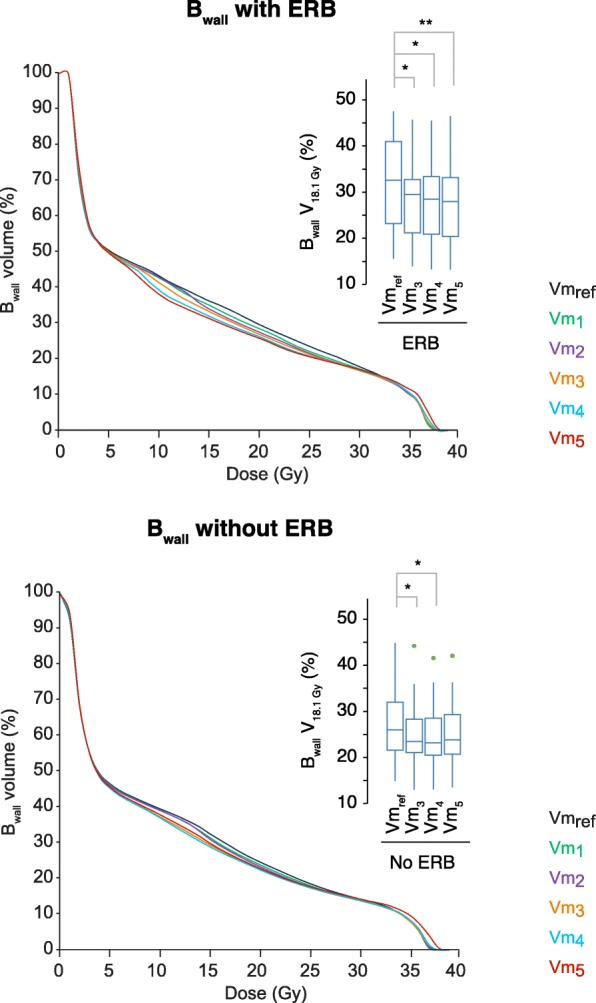
Dosimetric parameters for B_wall_. Median DVH from Vm_ref_ to Vm_5_ with ERB and without ERB. Box-and-whisker plots for Vm_ref_, Vm_3_, Vm_4_, and Vm_5_ of V_18.1 Gy_. Significant relations are shown with gray lines above the boxplots. * is set for P <  0.05 and ** for P <  0.005. Outliers are visible with green dots for out-values (1.5xIQR)

Figure 4 shows the B_wall_ DVH, with a focus on the V_18.1 Gy_, for plans from Vm_ref_ to Vm_5_ with and without ERB. The successive optimizations on R_wall_ slightly lowered V_18.1 Gy_ (*p* <  0.036) with and without ERB. A higher D_2%_ for F_heads_ is observed for plans with ERB (*p* <  0.01) (Table 2). No statistical differences in MU were observed for paired plans with and without ERB, MU increasing similarly with optimization. The fourth optimization Vm_4_ is found to be the best compromise between the R_wall_ sparing, PTV and uPRV homogeneity and dose coverage, and other OAR irradiation with and without ERB.

## Discussion

In this study, the potential for decreasing low and intermediate doses to the R_wall_ by pushing down the dose constraints to the R_wall_ in sequential plan optimization steps was investigated with and without ERB. Starting with individually optimized plans, the R_wall_ sparing could be improved in four optimization steps (Vm_4_) without impairing the PTV or uPRV coverage, although at the expense of more MU and slightly higher dose to the F_heads_. The delivery of more MU and its impact on the beam-on time could be solved by using flattening filter free beams (high dose-rate) that can reduce treatment delivery time. Three additional dose-constraints to the R_wall_ (Table 1), corresponding to the 90th percentile of the distribution of D_30%_, D_40%_, and D_med_ in Vm_4_ plans, can be proposed to improve the R_wall_ sparing in prostate SBRT as a consequence of this study: V_7.2Gy_ < 40%, V_13.1 Gy_ < 30%, and D_med_ < 6.5 Gy. This work was carried out for an SBRT dose prescription but a similar R_wall_ sparing could be achieved in conventionally fractionated prostate treatments.

By pushing down the R_wall_ DVO constraints to the limit (i.e. until the TPS is unable to optimize without impairing the PTV coverage) we observed that treatment plans with and without ERB gave similar results in the low-to-intermediate dose range. Patel et al. showed a dose reduction to the R_wall_ from using an ERB with 3-dimensional conformal RT (3DCRT) but not with intensity modulated RT (IMRT) [16]. Van Lin et al. reported similar results with the reduction of the R_wall_ mean dose, V_50Gy_, and V_70Gy_, with 3DCRT but not with IMRT [17]. However, Smeenk et al. found that the dose to the anorectal region was improved with an ERB, including for IMRT [18]. Two recent studies analyzed the effect of using an ERB for prostate SBRT in the range of intermediate to high doses. A linac-based study [19] found that the presence of the ERB increased the volume of the R_wall_ receiving high doses (V_95%_ and V_99%_) whereas no statistical difference was observed at an intermediate dose level (V_50%_). A Cyberknife-based study [20] observed that V_50%_, V_80%_, V_90%_ and V_100%_ were lower with an ERB.

In light of the findings of this study, it may be important for modulated delivery techniques to evaluate the dosimetric impact for the R_wall_ of the use of an ERB separately from the optimization process, which has an important influence on the dose to the OAR. Different optimization approaches may explain the conflicting results obtained in the studies above.

Avoiding the overlap of a target and an OAR in a beam projection is of critical importance for sparing healthy tissues with 3DCRT plans and direct planning software. With inverse planning techniques, convex isodoses and steep dose gradients reduce the importance of the proximity of targets and OAR for dose reduction. As reported by Kim et al., severe late rectal toxicity may be mostly correlated with the absolute volume of R_wall_ receiving high doses (>3cm^3^), but also with the circumference of R_wall_ receiving 39 Gy in 5 fractions (V_39Gy_ > 35%). Grade 2 acute rectal toxicity has been correlated with the circumference of R_wall_ receiving 24Gy in 5 fractions (V_24Gy_ > 50%) [7]. A recent study [8] showed that patients treated with magnetic resonance imaging (MRI)-based prostate delineation or an endorectal balloon (ERB) had favorable anorectal dose distributions (range of 5–60 Gy in 39 or 19 fractions) and favorable toxicity profiles. Van Lin et al. [21] had already observed less telangiectasia for patients with smaller volumes of R_wall_ exposed to doses >40Gy, corresponding to the group of patients with an ERB in their 3DCRT study. Based on these observations, it seems prudent to lower the intermediate doses to the R_wall_ by pushing down the DVO constraints on the R_wall_ regardless of the use or not of an ERB. To further decrease the highest doses received by the R_wall,_ while maintaining optimal target coverage, only the use of reabsorbable gel-spacers implanted between the anterior R_wall_ and the posterior aspect of the prostate gland before SBRT may help [12, 22, 23].

The number of patients selected for this dosimetric study, although small, was consistent with the previous studies for 3DCRT, IMRT and SBRT. Because the patients had repeated CT scans with and without ERB, a rigorous controlled comparison could be made. This is an important advantage compared to previous studies because an inflated ERB can modify the shape of the adjacent organs. The optimization strategy of reducing the dose to an organ, such as the R_wall_, in a stepwise manner while keeping the other constraints constant is comparable to a Pareto optimal front technique [24]. The preparation, by a single planner, of many plans per patient, is time-consuming and can potentially introduce planning bias [25]. Our methodology can be used as an alternative for centers that are not equipped with Pareto-surface based multicriteria optimization (MCO) planning software [26]. The interest of this kind of software is that it is fast and can contribute to removing manual planning bias.

## Conclusions

In conclusion, further optimization of the dose to the R_wall_, beyond the usual recommendations for SBRT of prostate cancer, was feasible without compromising dose homogeneity to the target (i.e., the PTV and the uPRV) with and without an ERB. Nonetheless, a larger amount of MU were required for the fully optimized plans. The main outcome of this work was to establish that one optimal technique for reducing the low- to- intermediate dose to the R_wall_ was a step wise optimization approach. Despite the inherent limitations of our study, we were unable to demonstrate that the use of an ERB allows additional low-to-intermediate dose reduction to R_wall_.

## Abbreviations

3DCRT: 3-dimensional conformal RT
B_wall_: bladder wall
CT_sim_: computed tomography simulation scan
D_med_: median dose
DSC: Dice similarity coefficient
DVH: dose-volume histogram
DVO: dose-volume optimization
D_xx%_: dose received by xx% of a volume
EQD2: equivalent dose in 2 Gy per fraction
ERB: endorectal balloon
F_heads_: femoral heads
HI_PTV_: homogeneity index for PTV
HIu_PRV_: homogeneity index for uPRV
IMRT: intensity modulated RT
MCO: multicriteria optimization
MRI: magnetic resonance imaging
MU: monitor units
OAR: organ at risk
PTV: planning treatment volume
R_wall_: rectal wall
SBRT: stereotactic body radiotherapy
TPS: treatment planning system
uPRV: urethral planning risk volume
Vm_1_ to Vm_5_: optimized plans in 5 sequential steps
VMAT: volumetric modulated arcs
Vm_ref_: reference plans
V_xx%_: volume encompassed by the xx% isodose line
